# Unusual Findings in Appendectomy Specimens of Adults: Retrospective Analyses of 1466 Patients and a Review of Literature

**DOI:** 10.5812/ircmj.12931

**Published:** 2014-02-04

**Authors:** Hakan Yabanoglu, Kenan Caliskan, Huseyin Ozgur Aytac, Emin Turk, Erdal Karagulle, Fazilet Kayaselcuk, Mehmet Akin Tarim

**Affiliations:** 1 Department of General Surgery, Faculty of Medicine, Baskent University, Ankara, Turkey; 2 Department of Pathology, Faculty of Medicine, Baskent University, Ankara, Turkey

**Keywords:** Appendectomy, Appendiceal Neoplasms, Enterobius, Entamoeba Histolytica

## Abstract

**Background:**

Diseases and tumors of the appendix vermiformis are very rare, except acute appendicitis.

**Objectives:**

This retrospective study was conducted to document the unusual findings in appendectomy specimens.

**Patients and Methods:**

Data of 1466 adult patients were gathered retrospectively. Appendectomy was performed in 1169 and in 297 patients following a diagnosis of acute appendicitis and during other abdominal operations, respectively. The data of 57 (3.88 %) patients who were pathologically reported to have unusual appendix findings were retrospectively collected. The records were analyzed according to patients’ age, gender, clinical presentations, operative reports, pathological reports and follow up.

**Results:**

Unusual pathologic examination findings were detected in the appendectomy specimens of 57 patients with a mean age of 48.34 ± 19. Twenty-nine patients (50.8 %) were male and 28 (49.2 %) were female. Normal appendix tissues were observed in specimens of 26 (45.6 %) patients and inflamed appendix in 31 (54.3 %). The most common unusual finding was parasitic diseases of the intestine. Pathological diagnosis of malignancy and benign features were reported in specimens of 14 and 43 patients, respectively. Macroscopic evaluation of appendectomy specimens during surgery might result in negligence of the presence of unusual pathology.

**Conclusions:**

Even if the macroscopic appearance of the specimen is normal or acute appendicitis, we suggest routine histopathological examination.

## 1. Background

Appendectomy is one of the most common surgical procedures done for acute appendicitis or during various abdominal operations. The lifetime incidence of acute appendicitis is 8.6% and 6.7 % for men and women, respectively (1). However, the incidence of appendectomy performed for various reasons is 12 % for men and 25 % for women (1). Most common cause of acute appendicitis is obstruction of the lumen. Although fecal impactions and lymphoid hyperplasia are the most frequent reasons of lumen obstruction, rare and unusual causes like intestinal parasites, radiocontrast agents, actinomycetes, endometriosis, tuberculosis, stromal tumors, carcinoid tumors, fruit pippins, adenomas, mucoceles, lymphomas, dysplastic changes, primary and secondary adenocarcinomas, eosinophilic granulomas, and granulomatous diseases may be seen (2-8).

## 2. Objectives

This retrospective study was designed to document the unusual findings in appendectomy specimens.

## 3. Materials and Methods

The data of adult patients who underwent appendectomy at Baskent University, Adana Teaching Hospital from January 1999 through February 2013 were analyzed retrospectively. This study was a retrospective clinical research study. During a 14-year period, patients who underwent appendectomy in our hospital were included regardless of the preoperative diagnosis. Specimens from patients operated at different centers and those examined by pathology department of our hospital were excluded from the study. The collected records were patients’ age, gender, clinical presentation, operative reports, pathological reports, and follow-up durations. The duration of follow-up was reported as months, beginning from the date of diagnosis until the last clinical information available up to February 2013. Histological reports were analyzed according to diagnosis and unusual findings were noted. The original pathology specimens with unusual findings were re-evaluated by an experienced pathologist (FK). This study was approved by Baskent University Institutional Review Board and supported by Baskent University Research Fund (KA13/50). Data were collected on a computer media and analyzed by SPSS software (SPSS Inc, Chicago, Illinois, united states).

## 4. Results

**Figure 1.**
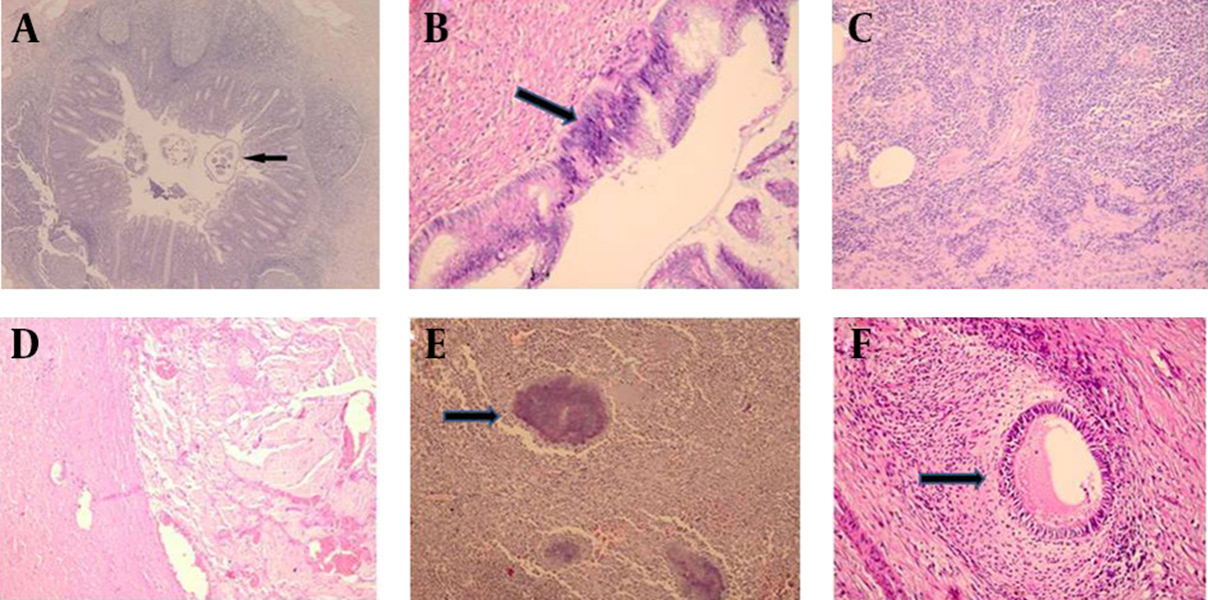
Unusual Histopathologic Findings **A:** Enterobius vermicularis image within the lumen of the appendix (black arrow), (HE, x40); **B:** High grade mucinous appendicial neoplasia (black arrow), (HE, x200); **C:** CD 79 a (+) in appendix, low grade lymphoma (HE, x200); **D:** Presence of mucine in wall of appendix (HE, x200); **E:** Actinomyces colonies in the appendix (black arrow), (HE, x200); **F:** Endometrial glands and stroma in the muscle layer of appendix wall (black arrow), (HE, x200).

Hospital records of 1466 patients who had underwent appendectomy (due to inflammatory or incidental reasons, either open or laparoscopic) were reviewed during the 14 years period beginning from January 1999 to February 2013. The data of 57 (3.88 %) patients who were pathologically reported to have unusual appendix findings were collected retrospectively. Of the 1466 patients who underwent appendectomy, 1169 were operated for acute appendicitis and the rest 297 were operated during other surgical procedures (gynecological cancers, laparotomies intending to acute abdomen, colon cancers, mesenteric ischemias, Amyand’s hernias, and etc.). Among these patients, 730 (49.7 %) were males and 736 (50.3 %) were females. Mean age of the patients was 36 ± 12 years ranging from 16 to 83 years. Out of 1466 cases, pathological evaluation of 328 (22 %) was normal. In 1138 cases (78 %), pathological reports were associated with acute inflammation showing changes of acute appendicitis (abscess, perforation, or gangrene). Unusual pathologic findings were detected in the appendectomy specimens of 57 patients with a mean age of 48 ± 19 years. Twenty-nine patients (50.8 %) were males and 28 (49.2 %) were females. Of these 57 specimens with unusual pathology, 26 (45.6 %) were non-inflamed and 31 (54.3 %) were inflamed (phlegmonous, perforated, or acute) appendix tissues. Eighteen (31.5 %) of the patients having normal histopathology were females and 8 (14 %) were males. On the other hand, 10 (17.5 %) of the patients who were reported to have inflamed appendix tissues were females and 21 (36.3 %) were males. Amongst 57 patients having unusual histopathologic findings, 14 had malignant (primary mucinous adenocarcinoma, neuroendocrine -carcinoid- tumours, lymphomas, or pseudomyxoma peritonei) and 43 had benign disorders. Most of the benign disorders were due to intestinal parasites. Of the 15 patients (57.6 %) presenting intestinal parasitosis, 12 had *Enterobious vermicularis* and three had Entamoeba histolytica (amebiasis). The etiological causations of unusual histopathologic findings in appendectomy specimens of 57 cases are summarized in Table 1. Some images of specimens of benign and malignant disorders are demonstrated in Figure 1. The clinicopathological characteristics of five patients with appendix mucinous adenocarcinoma and seven patients with neuroendocrine tumors (carcinoids) are summarized in Tables 2 and 3, respectively.

**Table 1.** Distribution of the 57 Cases Defined as ‘’Unusual Finding’’ According to Etiological Causes

	No. (%)
**Total Patients**	57 (3.88)
**Mucinous cystadenoma ( + mucocele)**	16 (1.09)
**Enterobius vermicularis**	15 (1.02)
**Neuroendocrine tumors**	7 (0.47)
**Mucinous adenocarcinoma**	5 (0.4)
**Amebiasis**	4 (0.27)
**Tubular adenoma**	2 (0.0 1)
**Lymphoma**	1 (< 0.01)
**Endometriosis**	1 (< 0.01)
**Actinomycosis**	1 (< 0.01)
**Pseudomyxoma peritonei**	1 (< 0.01)
**Foreign body granuloma**	1 (< 0.01)
**Focal severe dysplasia**	1 (< 0.01)
**Serrated adenoma**	1 (< 0.01)
**Hyperplastic poly**	1 (< 0.01)

**Table 2.** Clinicopathological Characteristics of the Five Patients With Primary Appendicular Mucinous Adenocarcinoma

Age (y)	Sex	Tumor size (cm)	Location	Treatment	Parietal Spread	Follow-up (Month)
**61**	F	4	Distal	Appendectomy + Right hemicolectomy	Mucosa	13
**47**	M	2	Base	Appendectomy + Right hemicolectomy	Mucosa	11
**71**	F	< 1	Distal	Appendectomy + Right hemicolectomy	Mucosa	12
**60**	F	< 1	Distal	Appendectomy	Serosa	3 (exitus)
**39**	F	2	Distal	Appendectomy + Right hemicolectomy	Serosa	4

**Table 3.** Clinicopathological Characteristics of the Seven Patients With Primary Appendicular Neuroendocrine Tumors (Carcinoid)

Age (y)	Sex	Tumor size (cm)	Location	Treatment	Parietal Spread	Follow-up (Month)
**56**	F	0.7	Distal	Appendectomy	Serosa	49
**23**	M	0.6	Distal	Appendectomy	Submucosa	63
**61**	F	0.6	Distal	Appendectomy	Serosa	2 (exitus)
**37**	M	1.1	Distal	Appendectomy + Right hemicolectomy	Serosa	42
**23**	F	0.7	Distal	Appendectomy	Submucosa	33
**29**	M	0.9	Distal	Appendectomy	Mucosa	18
**41**	M	0.8	Distal	Appendectomy	Submucosa	43

In those with diagnosis of mucinous adenocarcinoma, immediate right-sided hemicolectomy was performed for three patients synchronously with appendectomy by perioperative pathological frozen section confirmation and for one patient at a following session. A right-sided hemicolectomy at the second session was performed for another patient with neuroendocrine tumor invading the serosa. A patient for whom synchronous right-sided hemicolectomy was performed due to mucinous adenocarcinoma and another patient operated for rectum perforation secondary to metastasis of lung adenocarcinoma with a postoperative pathology diagnosis of mucinous adenocarcinoma in appendix had died. A patient who was diagnosed with mucinous adenocarcinoma and a previous metastatic colon carcinoma had died three months later. Another patient who was diagnosed with neuroendocrine tumor (carcinoid) with a previous history of cholangiocellular carcinoma had died two months later owing to multiple organ failure. Neither recurrence nor mortality had been seen after the mean follow up of 30 (4-63) months of the 12 patients that were reported to have primary malignant disease of the appendix. All patients with malignant tumors were diagnosed clinically with acute appendicitis, and none of them had symptoms of carcinoid syndrome or were preoperatively diagnosed with an appendicular tumors. After pathological confirmation of the diagnosis, abdominal ultrasonography, computed tomography (CT) scanning, and 24-hours urinary 5-hydroxyindoleacetic acid level measurements were performed for staging. Then, all patients were followed-up at the outpatient clinic every 3 or 6 months for the first year. Oral medications were prescribed to the patients that were found to have parasitic diseases of the intestine according to histopathological studies.

## 5. Discussion

Appendectomy is one of the most common surgical operations (9). Incidence of acute appendicitis is rather proportional with lymphoid development. It makes peak by the end of puberty and third decade of life. The gender ratio in acute appendicitis is about 1:1 prior to puberty. At puberty, male to female ratio becomes 2:1 (10). The most important causative factor for acute appendicitis is luminal obstruction. With the pathophysiological changes occurring due to luminal obstruction like continued mucus secretion, inflammatory exudation increasing intraluminal pressure, which obstructs lymphatic drainage and developing edema and mucosal ulceration, distension of appendix increases and results in venous obstruction. At the end of this process, ischemic necrosis occurs at the wall of appendix vermicularis (11). Fecaloids are the major factors resulting in luminal obstruction of appendix. There are many other rare reasons except this (2-8). Most prevalent unusual pathologic findings seen in appendectomy specimens after appendectomy due to any reason are parasitic diseases of the intestine and benign or malignant tumors (10). Enterobious vermicularis is the most common helminthic infestation agent of gastrointestinal tract in the world (12). The relation between Enterobious vermicularis and appendicitis was first described by Stil in the late 19th century (13). Although it is generally asymptomatic, its major symptom is pruritus ani. On the other hand, it may appear with serious complications like ileocolitis, enterocutaneous fistulas, urinary infections, mesenteric abscesses, salpingitis, and appendicitis. Incidence of Enterobious vermicularis existence in appendectomy specimens is between 0.6 % and 3.8 % (12, 14-19). Inflammation rate in appendectomy specimens that were infected with *Enterobious vermicularis* differs from 13 % to 37 % (20-22). Similarly, in our study 15 (1 %) patients had *Enterobious vermicularis* in their appendectomy specimens and of these, 3 (20 %) had shown inflammation related with acute appendicitis.

Appendicitis due to existence of *Entemoeba histolytica* is very rare and few cases are reported in literature (23-28). The certain frequency of this atypical presentation of parasitic disease is not known (29). It differs between 0.5 % and 2.3% among the limited number of studies in literature (15, 23, 30). In our study, *Entemoeba histolytica* was demonstrated in 4 (0.2 %) patients. Only in one (25 %) of these patients inflammation was shown histopathologically. Ratio of inflammation is very low in appendectomy specimens of patients determined to have both *Entemoeba histolytica* and *Enterobious vermicularis* (20-22). For this reason, negative laparotomy is seen more frequently in this group in contrast to all the other unusual pathological situations. Proper medications (oral metronidazole/pyrantel pamoate) were started after surgery for all the patients in this group. Neoplasms of the appendix are very uncommon and usually diagnosed at operation or autopsy. Malignant tumors of the appendix include carcinoids, lymphomas, mucoceles, primary adenocarcinomas, and mucinous cystadenocarcinomas. Benign tumors of the appendix consist of tubular adenomas, villous adenomas, leiomyomas, neuromas, and lipomas (3, 31, 32). In a study of Collins, investigating appendectomy specimens of 71000 patients operated for various clinical conditions, 958 (1.35 %) malignant and 3271 (4.6 %) benign tumors were determined (33). in our patients, we identified 30 (2 %) cases with malignant neoplasm in appendix specimens.

Neuroendocrine tumors (carcinoids) are the most common malignant tumors of appendix vermiformis (34). They are typically yellow-brown, small, hard and having limited surface tumors. They are generally diagnosed after appendectomies done for acute appendicitis or other surgical procedures with coincidence. In the series by Collins, carcinoids made up 51 % of the malignant tumors of the appendix (33). The reported incidence of appendix carcinoids in several studies ranges from 0.02 to 2.27 % of surgically removed appendixes (3, 9, 35-37). In Collins’s study, carcinoids were found in 0.7 % of all appendectomy specimens (33). Carcinoids were seen in 7 (0.47 %) patients in our study. All patients in our study had signs and symptoms of acute appendicitis. None of the patients had symptoms of carcinoid syndrome (flushing, diarrhea, cardiac symptoms, and bronchospasm). A large female preponderance is reported in all series (2-3:1) (38, 39). In many studies diameter of carcinoid tumors were found to be shorter than 1 cm and dominantly located at the tip of the appendix (3, 34, 40-42). While tumor localization in our study was concordant with the literature, incidence seemed to vary with gender (Table 3). The calculated risk of metastasis from tumors of 1 cm or smaller in diameter is nearly zero and therefore, can be managed with a simple appendectomy. Metastasis risk increases up to 85 % when the tumor diameter exceeds 2 cm. An appendiceal carcinoid tumor larger than 2 cm should be managed with a formal right-sided hemicolectomy (4, 14, 31, 34, 36, 41, 43-46). In our study, a right-sided hemicolectomy was performed to a patient with a carcinoid tumor of 11 mm in diameter invading serosa at a second session. No recurrence was observed after 42 months follow-up of.

Primary adenocarcinoma of the appendix is an extraordinarily rare tumor. It is defined in limited series in literature with an incidence of 0.08 % (33). This tumor is seen most commonly in patients between the age 50 and 55 years. Adenocarcinomas behave aggressively in a fashion similar to colonic adenocarcinomas; therefore, they must also be treated with the same aggressive approach (39). In our study, five patients had adenocarcinomas. Right-sided hemicolectomy were performed synchronous with appendectomy for three patients and for another patient after permanent pathological confirmation at a second session. No further surgical intervention was planned for one of the patients with metastatic disease who died at the third month of follow up due to multiple organ failure. Four patients are still alive without any recurrence and are still being followed up.

Mucinous cystadenoma is a rare tumor of the appendix associated with cystic dilatation, to which the more general term of mucocele has been applied. Incidence of mucocele in appendectomy specimens is between 0.2 % and 0.3 % (3). This ratio was found to be higher (1.09 %) than literature in our study. Mucocele generally behaves asymptomatic and is determined coincidentally during other abdominal operations. Mucoceles were found in five of our patients who were surgically explored for ovarian masses. However, mucoceles might be recognized clinically from features of acute appendicitis. Appendectomy is the standard of care for mucinous cystadenoma, whereas a cystadenocarcinoma requires a right-sided hemicolectomy. Because of the high association of mucinous cystadenoma with colonic and ovarian malignancies, follow-up with CT-scans, ultrasonography, and colonoscopic examinations must be performed during the postoperative period (3, 4, 31, 42, 47-49).

Endometriosis is defined as the presence of endometrial tissue outside uterine cavity (50). It rarely settles in gastrointestinal system. Intestinal endometriosis is classified as external endometriosis and occurs in only about 10 % of women with endometriosis. Appendiceal endometriosis is usually asymptomatic, but it occasionally causes appendicitis, perforation, and intussusceptions. The diagnosis of appendiceal endometriosis is based on the histological presence of endometrial tissue in the specimen. We found endometriosis in one of our patients’ appendectomy specimens. Rare pathogens like actinomycosis, lymphoma, and pseudomyxoma peritonei were found in very few ratios, which is in concordance with the reports in the medical literature. Our series have the highest frequency of unusual pathological findings in appendectomy specimens (3.88 %) among single center series in literature. Although there are some case reports in English medical literature, there are only few reports analyzing large series of patients (9, 51-54). However, the weaknesses of this study are its retrospective nature and including data only from a certain region of Turkey.

Although fecaloids and lymphatic hyperplasia are the foremost reasons of acute appendicitis, rare conditions must not be neglected. Otherwise, pure macroscopic assessments of the specimens without histopathologic confirmation may cause overlooking unusual reasons that result in incomplete surgical and medical treatments. This situation, especially with the existence of malignant tumors, brings important medical, social, ethical, and legal problems. Hence, we conclude that all the appendectomy specimens must be examined histopathologically independent from macroscopic aspect.
